# Annotations

**Published:** 1895-05-18

**Authors:** 


### Annotations.

### The Moral of the Cab-call.

Among the ingenious writers for the medical press who periodically scare the public by laying bare fresh paths by which infection may be spread, it seems an axiom that not only may it pass by kissing and such like crude modes of personal contact, but that a whole host of intermediate substances, animal, vegetable, and mineral, may serve as vehicles by which infective particles may jump the gap between one patient and another. We hear of scarlet fever being disseminated by milk, of typhoid being broadcast by water, of the slates on which the children in small day schools tot up their little sums spreading infection from child to child by virtue of the sequence of salivas by which the slates are washed. Nay, our law and our religion are included in the same indictment. Kissing the Book and partaking of the Communion cup are openly assailed as fraught with danger of infection. The cynic smiles, and sarcastically asks why any of us are yet alive. And yet these methods of infection are obviously possible. We have all heard of cholera being conveyed by flies. In truth, the possibility of this mode of conveyance of disease has been practically proved by recent observations in India, and was demonstrated at the recent soirée of the Royal Society. These things, then, being accepted, let us call attention to another possible mode of infection, viz., the cab whistle, which hangs upon the hat stand in the hall. The master speeding the parting guest, the housemaids, the "young gentlemen," the butler, and the "buttons," all alike place this noisy instrument between their lips and clearly run a risk of mutual infection. This instrument obviously should be a much more efficacious means of spreading disease than several we have mentioned, and the question at once arises, is disease ever spread in that way, and can anyone point to a case in which it has been so caught? The proofs which are being accumulated that infection is everywhere, and that we stand in peril all our lives, so far from proving the importance of infection *per se* tend rather to show that it alone is not the *causa causans* of disease. We are getting somewhat tired of microbes, but we badly want to know why they so often do not take root, for it is probable that the key to complete knowledge of infectious diseases will be found in the study, not of infection, but of immunity.

### A Toothache Benefactor.

Who shall sing the praises of William Watson Mackay! Unknown to fame, he is yet the benefactor of many. By a deed of gift made in 1890 he assigned to trustees the sum of £500, the income of which is to be devoted to the payment of a doctor or of doctors who will extract the teeth of the poor of the town of Kirkintilloch. Mr. Mackay explains that, besides having a general regard for the welfare of his fellow-men, he has been in the habit of gratuitously extracting the diseased or malformed teeth of any applicant, and wishes the service to be continued beyond his own life. He specially desires that the patient may be given to understand that the tooth-drawing is not a charity, but "an act of gracious, loving regard." Alas! it is difficult for us to realise that our dentist loves us. We are nearly as instinctively sceptical as Izaak Walton's worm. Nevertheless the people of Kirkintilloch have not been slow to avail themselves of Mr. Mackay's beneficence. Two doctors have been appointed by the trustees to operate upon the jaws of Kirkintilloch, and the number of extractions averages about a thousand per annum, and promises to increase rather than diminish. The result is that the doctors begin rather to regret their appointment. The remuneration intended by the donor of the bequest was a shilling per tooth, but as the income derivable from £500 invested at 4 per cent. is only £20, it results that the pay is only about fourpence a tooth. If, however, in any year fewer than 400 extractions take place the trustees pay only for the teeth extracted at the rate of one shilling per tooth; but when the number exceeds the 400 no extra remuneration is given. This means, in face of the increasing stream of sufferers, that three-fifths of their work is done gratuitously, and they feel that they, as much as Mr. Mackay, deserve to be reckoned as the benefactors of Kirkintilloch. But at least the bequest is probably unique in the history of legacies.

### A Bristol Philosopher, or What?

In times by no means remote every occupation, and even every amusement and dilettantism, had its own technical terms, to which mere outsiders paid becoming respect. What man of forty-five has not studied, for example, the "Language of Flowers" in the days of his "calf love"? In like manner, how few women there are even of the present day who do not know something of the "Language of Palmistry"? But poor medicine, albeit of necessity the most technological of all things technical, is now in bad odour with a Bristol correspondent, and presumably, therefore, with many other "plain and downright" men and women, because it does not use English words and words of one syllable only in its descriptions and delineations of structure, functions, diseases, and treatment, when dealing with the human body and its manifold ills. This, it seems to us, is really going a little too far. Perhaps it would be considered by most people a sufficient answer to the question why we do not use simple English terminology in all our writings, to say, that "we do not because we cannot." No doubt in the "foolish old times" of medicine, when an appearance of learning and profundity was sought after by medical writers, because such an appearance really imposed upon the general public and the half-taught or wholly untaught members of the medical rank and file, many stupid and barbarous terms were brought in which a more honest and cultivated taste would have scorned to use. But in our times one of the surest and shortest of all the roads to contempt is a pretence of knowledge which is not justified by possession. As a matter of experience, therefore, the truth is, that the best medical writers do make every reasonable attempt to produce books and articles which shall be as simple and pure in their literature as they are in their science. But when all is done, does it not remain true that medicine in its widest aspects is a science and an art? And must not every science and art have terms which belong to it exclusively and not to other sciences and arts, or even to every-day speech? As well might we say that a church should not have a steeple, because common houses do not have steeples, as say that medicine must not have its own terminology, because the common language of every-day life does not require a special terminology. The Bristol correspondent is certainly not a philosopher. But, if not a philosopher, what is he?

